# Information Needs of Patients About Immunosuppressive Medication in a German Kidney Transplant Sample: Prevalence and Correlates

**DOI:** 10.3389/fpsyt.2019.00444

**Published:** 2019-06-28

**Authors:** Felix Klewitz, Mariel Nöhre, Maximilian Bauer-Hohmann, Uwe Tegtbur, Lena Schiffer, Lars Pape, Mario Schiffer, Martina de Zwaan

**Affiliations:** ^1^Department of Psychosomatic Medicine and Psychotherapy, Hannover Medical School, Hannover, Germany; ^2^Project Kidney Transplantation 360° (NTX 360°), Hannover Medical School, Hannover, Germany; ^3^Department of Sports Medicine, Hannover Medical School, Hannover, Germany; ^4^Department of Nephrology and Hypertension, Hannover Medical School, Hannover, Germany; ^5^Department of Pediatric Kidney, Liver and Metabolic Diseases, Hannover Medical School, Hannover, Germany; ^6^Department of Nephrology and Hypertension, University Hospital Erlangen, Erlangen, Germany

**Keywords:** Satisfaction with Information about Medicines Scale, adherence, kidney transplantation, immunosuppressive medication, information needs of kidney transplant recipients

## Abstract

**Background:** Worldwide clinical guidelines for the care of kidney transplant (KT) recipients recognize the importance of health care providers imparting appropriate immunosuppressive medication (ISM) information for the facilitation of safe medication self-management. The extent of medication information made available is, however, not necessarily what patients require to know about their prescribed medicines. A useful indicator for determining the quality of prescription practice is to what degree the provided information meets the personal needs of patients. No previous studies have focused on the ISM information needs of KT patients. This study aims to investigate how satisfied KT patients are with the provided ISM information and to examine the association between satisfaction levels and socio-demographic, psychosocial, and transplant-related variables.

**Materials and Methods:** KT patients (*n* = 440) were asked to complete a series of self-report questionnaires to evaluate the variables adherence, ISM experience, perceived social support, symptoms of anxiety, and depression, and transplant-related information (e.g., donation type). ISM information needs were assessed with the Satisfaction with Information about Medicines Scale (SIMS-D).

**Results:** On average, 35.9% of the answers to the SIMS-D items indicated dissatisfaction with the received information; dissatisfaction was more prevalent for the SIMS-D subscale “potential problems” (46.1%) than the SIMS-D subscale “action and usage” (26.7%). On an individual item level, the dissatisfaction with information concerning ISM side effects on drowsiness (57.1%) and sex life (56.3%) was most notable. Higher satisfaction with ISM information was correlated with higher age, better adherence, higher perceived social support, and lower anxiety levels. Multiple linear regression analyses revealed that adherence, perceived social support, and age were independently associated with ISM information satisfaction. No associations were found with sex, educational level, partnership status, symptoms of depression, experience of side effects, and transplant-related variables.

**Discussion:** The data indicate that a substantial proportion of KT patients have unmet ISM information needs, especially with regard to potential problems of ISM. Dissatisfaction with ISM information is a potential amendable risk factor for KT patients engaging in non-adherent behavior, thus justifying further research in this area. ISM information should be tailored to meet the individual needs of KT patients in order to promote optimal medication self-management and adherence behavior.

## Introduction

Chronic kidney disease (CKD) constitutes a rapidly growing global health problem (1). In the end-stage of CKD, kidney transplantation (KTx) is considered to be the therapy of choice (2, 3), since it is significantly linked to reduced mortality and morbidity when compared to dialysis (4). One of the main long-term challenges that this treatment form entails is decreased kidney transplant (KT) function over time and the occurrence of acute or chronic rejection episodes that contribute to renal allograft loss (4, 5). Medically this challenge is met by placing KT recipients on a life-long therapy plan of immunosuppressive medication (ISM) (4). Central to the success of the therapy plan is adherence behavior, which the World Health Organization (WHO) defines as “the extent to which a person’s behavior … corresponds with agreed recommendations from a health care provider” (6).

Adherence is considered to be multifactorially determined (4, 6). Provision of medication information has been recognized by worldwide KTx clinical guidelines as one key factor for the facilitation of safe medication self-management and of adherence (4, 7). Information is especially crucial for patients receiving polypharmacy, as demonstrated by Wu et al. (8): regular counselling by pharmacists was associated with reducing the mortality risk by 41% in patients taking medicines for chronic conditions. ISM in particular requires a complex drug regimen, which is far from self-explanatory. Adequate ISM knowledge (e.g., schedule, managing possible side effects, etc.) thus forms the bedrock of successfully handling the long-term therapy plan agreed upon with the relevant health care providers. As Bertram et al. (9) fittingly phrased, “How can you be adherent if you don’t know how?” Health care providers are accordingly advised by worldwide KTx clinical practice guidelines to provide appropriate information about treatment and prescribed medicines (4, 7). From a legal perspective, this standard has been embedded for all illnesses in the German Patients’ Rights Act in 2013 (10). Relevant literature on this subject observes, however, substantial knowledge gaps in patients taking medicines regularly. In the study by Romero-Sanchez et al. (11), 71.9% of patients (*n* = 7,287) acquiring medication with or without prescription were considered to have inadequate knowledge about their medication. This seems to be a long documented problem, with studies reporting medication knowledge gaps (e.g., medication purpose, lifestyle changes, dosage, etc.) of patients, who have recently been discharged from the hospital, as far back as 1998 (12–14). Medication knowledge is, however, heterogeneously operationalized and other studies with different methodology show patients to be more proficient in this area, e.g., adult patients were able to adequately recall 86% of the medical information provided by their physician (15). In the case of CKD, the majority of patients feel ill-informed about treatment modality and initiation (e.g., risks and burdens) by their physicians (16), which is substantiated by a related review (17). Other information sources seem to be unable to bridge these deficits: available information leaflets in the UK about CKD are judged to be difficult to understand, incomplete, and lacking in quality (18). CKD patients having to adhere to phosphate-binding medication seem to require more information about their medication than has been provided to them (19). When it comes to medication knowledge deemed critical for appropriate ISM usage, a sample of *n* = 239 KT patients answered only 70.1% of multiple-choice questions correctly, which is considered to be insufficient from a clinical perspective (9).

A one-size-fits-all approach to the information provision problem seems hardly promising: patients differ in their coping styles and subsequently vary in how they respond to the trajectory of a disease and the treatments involved (20). The unique approach to any given disease may thus impel patients to seek different types and detail of information (19–21). As Weinman (20) illustrates, some patients adopting an avoidant coping style may experience distress when information is too detailed, whereas other patients who want to actively participate in the treatment process may be optimally supported by receiving elaborate information about, e.g., adverse effects of medication (19). In line with this notion, Berry et al. (22) were able to demonstrate that different types of side effect description (qualitative vs. quantitative) in medication information leaflets led study participants (*n* = 750) to substantially overestimate the risk of developing side effects in the qualitative condition. The quality of good prescription practice should thus not only be considered from the perspective of what patients actually know, but also take into account how satisfied patients are with the received information (21).

To our knowledge, little related literature is available about how satisfied KT patients are with information about ISM (23, 24). Concerning associated factors, there is no information available in the literature for KT patients; however, information can be deduced, e.g., from HIV patients who have to manage an equally complex treatment regimen: Gellaitry et al. (25) reported an association between dissatisfaction with information about highly active antiretroviral therapy and lower adherence behavior. A recent review investigating depression in the KTx population concludes that KT recipients have lower rates of depression than CKD patients receiving alternative renal replacement therapies; however, the prevalence rates of the former were still higher compared to the general population (26). Depression has been found to adversely impact clinical outcomes (e.g., cardiovascular mortality) and behavioral dimensions (notably adherence) in this population (26), thus making psychosocial variables an area of interest. Another variable worth considering is social support, since friends and family can be a substantial source of information when it comes to other continuous prescription medication (27). Finally some health care providers are hesitant to discuss medication side effects in detail fearing disadvantageous treatment consequences (28). It should therefore be worthwhile to explore how feeling informed about ISM relates to the actual experience of side effects.

The primary aim of the present study was to investigate satisfaction with information received about ISM among patients after KTx. In addition, the association between satisfaction with ISM information and sociodemographic, transplant-related variables, adherence, perceived social support, ISM experience (e.g., side effects) and symptoms of anxiety, and depression was explored. We employed the Satisfaction with Information about Medicines Scale (SIMS) that was developed by Horne et al. (21) as a valid measure to assess how satisfied patients are with information received about medication. This questionnaire can be used to identify specific unmet and over-met information needs. It has been applied in studies with patients suffering from various illnesses in different cultural contexts (19, 21, 29–37), but not in a KT sample.

## Materials and Methods

### Study Design

This cohort study had a cross-sectional design with an explorative approach. Data were obtained within the Innovationsfond project “Kidney Transplantation 360°” (2). KTx 360° seeks to establish an organized post-transplant care structure in order to improve follow up-care for KT patients by integrating relevant medical fields and optimizing the collaboration between transplant centers and the nephrologists operating in local private practices (2).

### Sample

From May 2017 to July 2018, 957 KT patients (age ≥ 16) were approached and 440 KT patients (46%) participated. Study participants were significantly younger (51 versus 53 years; Mann–Whitney *U Z*-score = −2.055; *p* = 0.040) and had a significantly shorter time since KTx (4 versus 7 years; Mann–Whitney *U Z*-score = −8.651, *p* < 0.001) than the approached patients who did not participate. Age and time passed since KTx were significantly and positively correlated (*r* = 0.166; *p* < 0.001). When looking at the distribution of donation type, the percentage of living donor recipients was significantly higher in the participants compared to the nonparticipants (55.9% versus 44.1%; χ²(1) = 9.318; *p* = 0.002). There were no significant differences in sex and pre-KTx dialysis duration. KT patients who were undergoing dialysis treatment or who had severe cognitive disabilities hindering them to fill out the questionnaires were excluded from the study. The ethics committee of the Hannover Medical School approved the study (3464-2017), and written informed consent was given by all participating KT patients.

### Measures

#### Satisfaction With Information About Medicines Scale (German version, SIMS-D)

**Figure 1:**
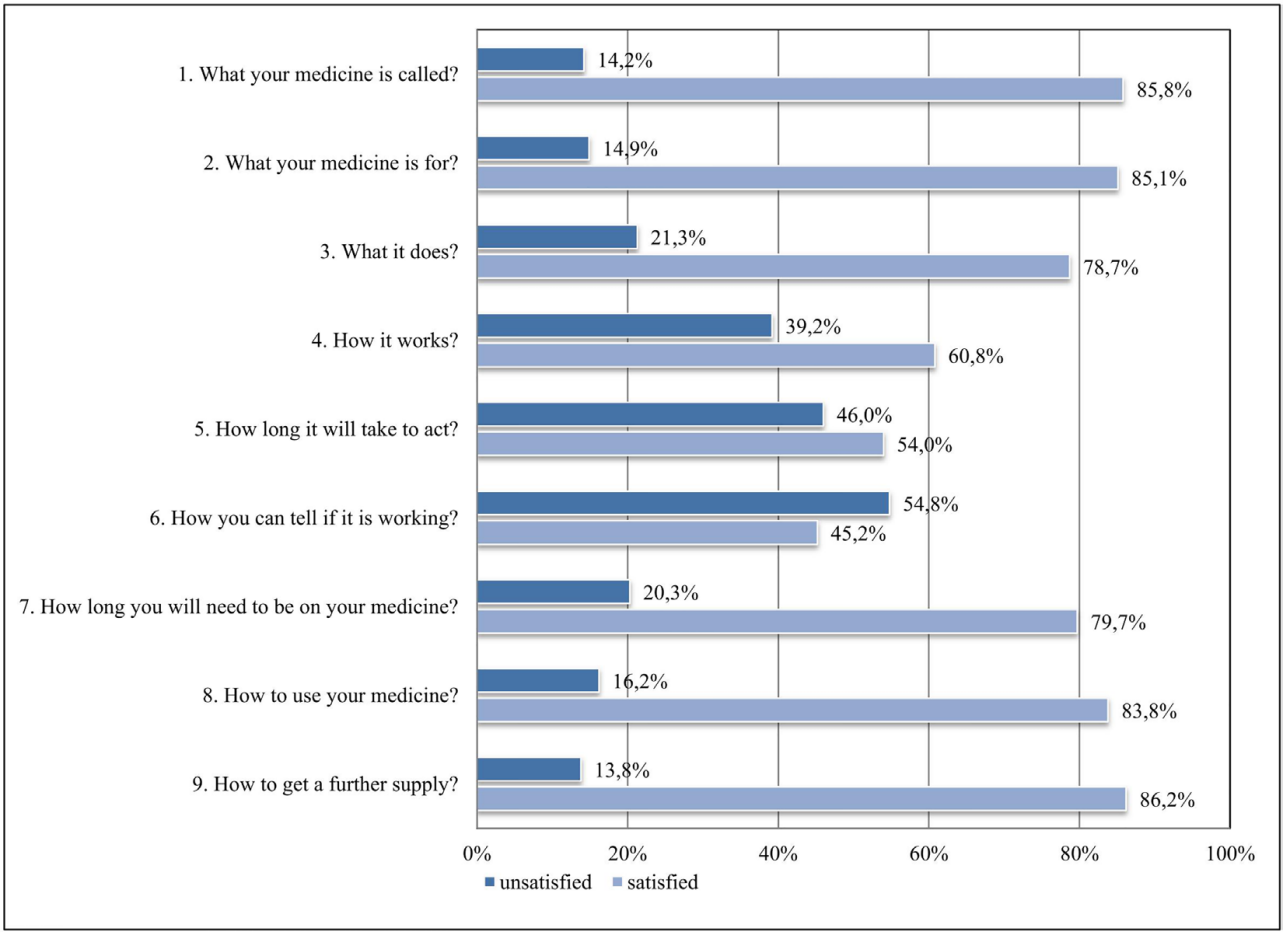
Satisfaction with information about “action and usage” of immunosuppressive medication (ISM).

**Figure 2:**
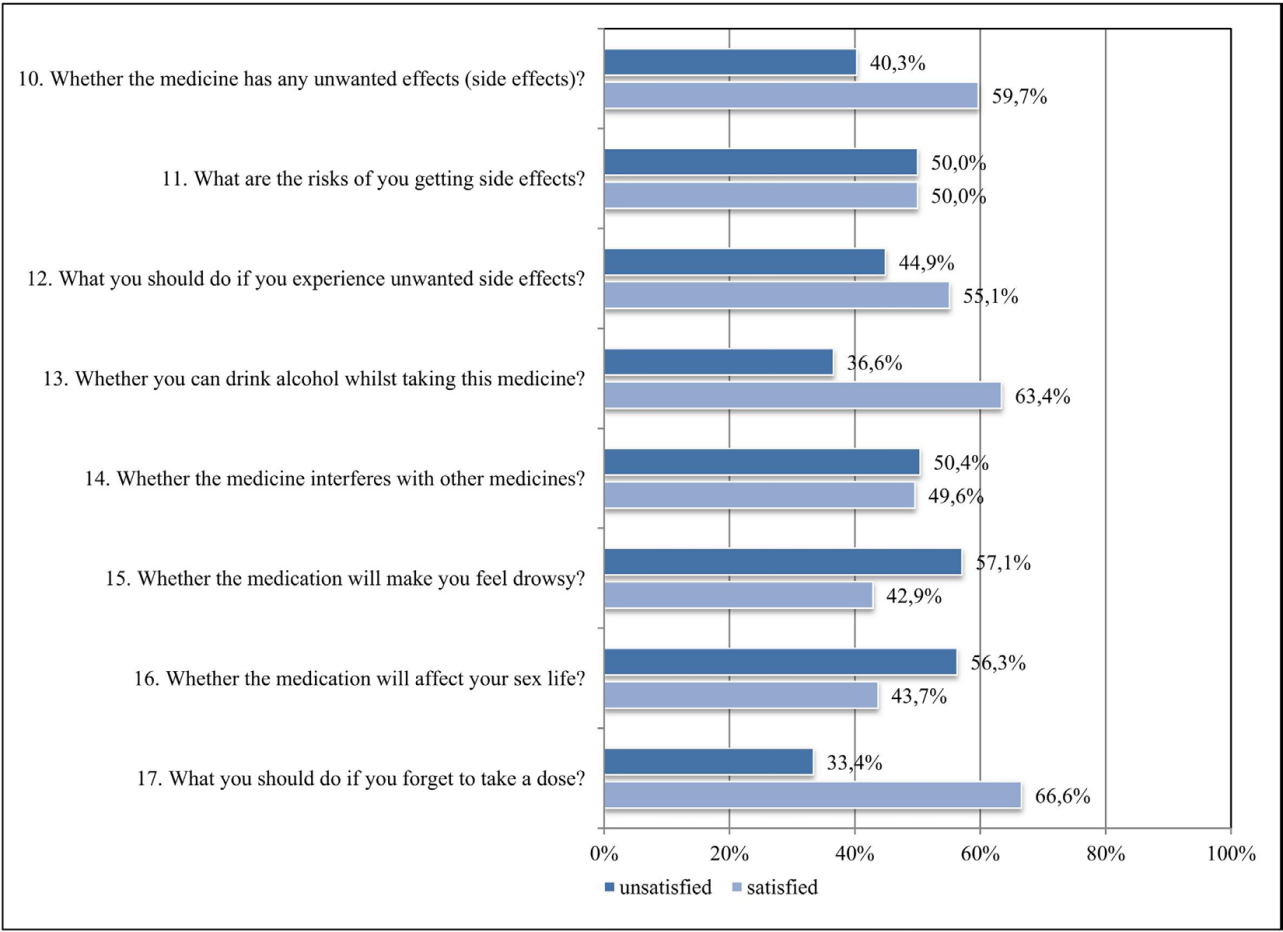
Satisfaction with information about “potential problems” of ISM.

The SIMS-D assesses patients’ satisfaction with 17 topics of information considered essential for safe and accurate self-management of medicines according to the recommendations of the Association of the British Pharmaceutical Industry (21). Nine items refer to the information received about “action and usage” of the medication (e.g., “what it does”; **Figure 1**), and eight items refer to information received about “potential problems” of the medication (e.g., “what are the risks of you getting side effects”; **Figure 2**). For each item, patients indicate if the information they have received is “too much,” “about right,” “too little,” “none received,” or “none needed.” Reports of “about right” and “none needed” are classified as satisfied and receive a score of 1. The remaining answering options are classified as dissatisfied and are scored as 0. The scores are summed up to obtain a satisfaction rating for the total scale ranging from 0 to 17 and for each subscale ranging from 0 to 9 for “action and usage” and from 0 to 8 for “potential problems.” Higher summary scores indicate a higher degree of satisfaction with information received. The SIMS-D was translated into German and validated in a sample of 264 chronically ill patients (31). For this study, patients were asked to only consider ISM in their rating; the SIMS-D was adapted accordingly with the approval of the original authors. Cronbach’s α was 0.906 for the SIMS-D total score, 0.833 for the subscale “action and usage,” and 0.878 for the subscale “potential problems.”

#### Medication Adherence Rating Scale (German version, MARS-D)

The MARS-D is a five-item self-report instrument focusing on non-adherent behavior (e.g., altering the dose of medication) on a five-point scale (5 = “never,” 4 = “rarely,” 3 = “sometimes,” 2 = “often,” and 1 = “very often”) (38). Scores range from 5 to 25 with higher scores indicating higher adherence. In line with previous work (39, 40) KT patients were considered to be non-adherent if they scored less than 25. The MARS-D was translated into German and validated in a sample of 523 patients with “chronic diseases and patients with risk factors of cardiovascular disease” (41). For our study, patients were asked to only consider ISM in their rating; the MARS-D was adapted accordingly with the approval of the original authors. Cronbach’s α for the total score was 0.694.

#### Hospital Anxiety and Depression Scale (German version, HADS-D)

Anxiety and depression were measured with the HADS-D (42), an internationally widely used, reliable, and valid self-report instrument for the assessment of levels of anxiety and depression in physically ill patients [German version by Herrmann et al. (43)]. Each of the two subscales consists of seven items, which are rated on a four-point Likert scale resulting in a sum score ranging from 0 to 21 for each subscale. Higher scores indicate higher levels of depressive or anxiety-related symptoms. Scores ≥ 11 are indicative for clinically relevant symptoms of anxiety and depression (42). Cronbach’s α in our sample was 0.862 for the depression subscale and 0.815 for the anxiety subscale.

#### Perceived Social Support (F-SozU K7)

Perceived social support was assessed with the seven-item short form of the German F-SozU K-7 (44, 45). Patients were asked to rate seven items pertaining to anticipated and perceived social support on a five-point Likert scale, ranging from 1 (“does not apply”) to 5 (“exactly applicable”). Total scores range from 7 to 35 with higher scores being indicative of higher perceived social support. Cronbach’s α in our sample was 0.911.

#### Medication Experience Scale for Immunosuppressants (MESI)

The German MESI is a seven-item self-report questionnaire that assesses subjective experiences and attitudes of patients concerning ISM (46). In items 1–3, patients are asked to rate their subjective experiences with the side effects of their ISM on a six-point Likert scale (0 = no side effect, 1 = trivial, 2 = mild, 3 = moderate, 4 = marked 5 = severe). Items 4–7 assess patients’ cognitive beliefs and knowledge about the side effects of their ISM on a five-point Likert scale [answering options vary in accordance with the items; for a detailed description, see Ref. (46)]. Total scores range from 4 to 33 and higher scores reflect more negative attitudes and experiences with side effects of ISM. Although not an explicit cut-off point, scores > 15 indicate that patients’ adherence might be at risk (46). Cronbach’s α in our sample was 0.736.

#### Demographic and Clinical Details

Sociodemographic characteristics and medical data including sex, age (years), level of education (≤12 years/>12 years), partnership status (yes/no), donation type (living/deceased donor), time passed since KTx (months), pre-KTx dialysis treatment (yes/no), and pre-KTx dialysis duration (in months if applicable) were assessed using a self-report questionnaire or were taken from the patient charts.

### Statistical Analysis

For each variable descriptive statistics (percentage, median with 25–75% interquartile ranges (IQR), mean and standard deviation) were calculated accordingly. Pearson’s correlations were performed for SIMS-D and ordinal/metric variables (F-SozU K7, MESI, HADS-D, age, pre-KTx dialysis duration, time passed since KTx, MARS-D total score). Furthermore Mann–Whitney *U* tests were utilized to calculate differences in SIMS-D scores between two groups (sex, level of education, partnership status, pre-KTx dialysis treatment, and donation type. Eta squared (η^2^) was used as a measure of effect size to discern the proportion of variance in SIMS-D scores accounted for by the selected variables. Multiple linear regression analyses were performed with the SIMS-D total and the two subscales as the dependent variables. Sociodemographic variables (sex, age, and educational level) and variables that were significantly associated in the correlation analysis were defined as the independent variables. Statistical significance was set at *p* < 0.05. All statistical analyses were performed using IBM^®^ Statistical Software Package of Social Science (SPSS^®^, Chicago, IL, USA) version 25.

## Results

### Sample Characteristics

Of the first 440 consecutive KT patients included in NTX360°, a total of 397 (90.2%) completed the SIMS-D without any missing data.

### Descriptive Statistics

**Table 1** summarizes demographic and clinical details of the sample. The median age of the cohort was 51 years; slightly more men (59.4%) participated than women (40.6%). Most participants had received less than 12 years of formal education (85.3%). Time passed since KTx amounted to a median of 53 months. Most KT patients had undergone pre-KTx dialysis treatment (88.2%) with a median duration of 61 months. The majority of KT patients had received a donor kidney from a deceased donor (68.0%) in comparison to a living donor (32.0%). Overall, 67.8% reported to be in a partnership.

**Table 1:** Sociodemographic and clinical characteristics.

Characteristics	N	Mean (SD)	Median (IQR)
Age (years)	397	51 (15)	53 (20)
Time passed since KTx (months)	397	53 (56)	41 (65)
Pre-KTx dialysis duration (months)	390	61 (49)	53 (79)
	N	%	
Sex			
Female	161	40.6%	
Male	236	59.4%	
Pre-KTx dialysis			
Yes	344	88.2%	
No	46	11.8%	
Partnership			
Yes	269	67.8%	
No	128	32.2%	
Donation type			
Living donor	127	32.0%	
Deceased donor	270	68.0%	
Level of education			
≤12 years	326	85.3%	
>12 years	56	14.7%	

SD, standard deviation; IQR, interquartile range.

**Table 2** gives a detailed description of the SIMS-D answer distributions (total score and both subscale scores). On average, 35.9% of the answers to the rated SIMS-D items indicated dissatisfaction with the received information; it should be noted that only 5% of the total reported dissatisfaction was due to perceived excessive information about ISM; 19.4% of the patients (*n* = 77) reported being completely satisfied with the information received about all the ISM topics, while 1.8% of KT patients (*n* = 7) reported dissatisfaction across all SIMS-D items.

**Table 2:** Overall results of the Satisfaction with Information about Medicines Scale (SIMS-D).

Scales	SIMS-D answers indicating dissatisfaction (%)			SIMS-D answers indicating satisfaction (%)
	None received	Too little information	Too much information	
SIMS-D “action and usage” subscale	5.8%	14.8%	6.1%	73.3%
SIMS-D “potential problems” subscale	14.4%	27.9%	3.8%	53.9%
SIMS-D total	9.9%	21%	5%	64.1%
	Aggregate dissatisfaction (%)			Aggregate satisfaction (%)
SIMS-D “action and usage” subscale	26.7%			73.3%
SIMS-D “potential problems” subscale	46.1%			53.9%
SIMS-D total	35.9%			64.1%

**Figure 1** exemplifies the variation of answers on an individual item level for the SIMS-D “action and usage” subscale. While more than three quarters of KT patients were generally quite satisfied with information concerning what their ISM is called, what it is for, what it does, duration of ISM treatment, how to use it, and how to get a further supply, more than half were dissatisfied about how to tell if ISM is working. Also 46% were dissatisfied with ISM information about “how long it will take to act” and 39.2% were dissatisfied with ISM information about the topic “how it works.” On average, 26.7% of the answers to the items of the SIMS-D subscale “action and usage” indicated dissatisfaction with ISM information.

**Figure 2** illustrates the distribution of answers for the SIMS-D subscale “potential problems” of ISM. KT patients were most dissatisfied with information about ISM causing drowsiness (57.1%) and affecting sex life (56.3%). About half of the sample was dissatisfied with ISM information they had received about the risks of experiencing side effects and whether the ISM “interferes with other medicines.” KT patients seemed to be more satisfied with ISM topics about what to do when missing a dose (66.6%), drinking alcohol (63.4%), the general occurrence of side effects (59.7%), and what to do when experiencing side effects (55.1%). On average, 46.1% of the answers to the items of the SIMS-D subscale “potential problems” indicated dissatisfaction with ISM information.

### SIMS-D Associations With Other Clinical Variables

#### Medication Adherence and Satisfaction with ISM Information

There was a significant positive correlation between the SIMS-D (total score and both subscale scores) and the MARS-D score with small effect sizes (**Table 3**). That is, KT patients with higher levels of ISM information satisfaction had higher adherence ratings. After dichotomizing the MARS-D score (total score <25 = non-adherent), more than a third of the sample were classified as non-adherent to their ISM (39.8%).

**Table 3:** Correlational analyses of the SIMS-D scales.

Correlational analysis	N	SIMS-D total		SIMS-D action and usage		SIMS-D potential problems	
		r	p	r	p	r	p
Age (years)	397	0.127*	0.011	0.150**	0.003	0.086	0.087
Time passed since KTx (months)	397	0.036	0.472	0.058	0.245	0.011	0.826
Pre-KTx dialysis duration (months)	390	0.020	0.691	0.032	0.528	0.006	0.898
MARS-D total score	394	0.168**	0.001	0.175**	<0.001	0.134**	0.008
HADS-D depression subscale	395	−0.082	0.106	−0.059	0.239	−0.086	0.086
HADS-D anxiety subscale	393	−0.133**	0.008	−0.118*	0.019	−0.124*	0.014
MESI	352	−0.069	0.196	−0.054	0.308	−0.070	0.192
F-SozU K7	395	0.184**	<0.001	0.144**	0.004	0.187**	<0.001

*p < 0.05; **p < 0.01

F-SozU K7, Questionnaire for Perceived Social Support; HADS-D, Hospital Anxiety and Depression Scale; MARS-D, Medication Adherence Rating Scale; MESI, Medication Experience Scale for Immunosuppressants.

#### Perceived Social Support and Satisfaction With ISM Information

A significant positive correlation with small effect sizes was found between perceived social support and levels of ISM information satisfaction for the SIMS-D total score and both SIMS-D subscale scores (**Table 3**). Specifically, KT patients with higher perceived social support were also more satisfied with the received ISM information.

#### ISM Medication Experience and Satisfaction With ISM Information

The average MESI score in our sample was 14.83 and 46.9% of the KT patients had MESI scores > 15. No significant correlation was detected between the MESI score and the SIMS-D total score and both SIMS-D subscale scores.

#### Depression and Anxiety and Satisfaction With ISM Information

In accordance with the recommended cut-off value, 9.6% of the KT patients were experiencing clinically relevant depressive symptoms and 12.2% clinically relevant anxiety-related symptoms. The HADS-D anxiety score correlated negatively with the SIMS-D total score and both SIMS-D subscale scores with small effect sizes (**Table 3**), meaning that KT patients experiencing more anxiety symptoms were also less satisfied with the received ISM information. No correlation was observed between SIMS-D scores and the HADS-D depressions score.

#### Differences in Satisfaction With Information About ISM Across Sociodemographic and Transplant-Related Clinical Variables

A statistically significant correlation was found between age and the SIMS-D total score and the SIMS-D subscale score “action and usage” (**Table 3**). Older patients seemed to be more satisfied with the information they had received about ISM than younger patients. No correlation was found for the SIMS-D subscale score “potential problems” and age. Furthermore, no statistically significant correlations were detected between the SIMS-D scores and the variables sex, donation type, level of education, pre-KTx dialysis treatment, partnership status, pre-KTx dialysis duration, and time passed since KTx (**Table 4**).

**Table 4:** Comparison of SIMS-D scale scores between dichotomous variables.

	N	SIMS-D total		SIMS-D action and usage		SIMS-D potential problems	
		Median (IQR)	Statistics *U*-tests	Median (IQR)	Statistics *U*-tests	Median (IQR)	Statistics *U*-tests
Sex							
Female	161	11.00 (9)	*Z* = −.346,	7.00 (4)	*Z* = −.604,	4.00 (5)	*Z* = −.657,
Male	236	11.00 (9)	*p* = .729, η^2^ = 0	7.00 (4)	p = .546, η^2^ = .001	4.00 (6)	*p* = .511, η^2^ = .001
Level of education							
≤12 years	326	11.00 (9)	*Z* = −.387,	7.00 (4)	Z = −.125,	4.00 (6)	Z = −.168,
>12 years	56	11.00 (8)	*p* = .699, η^2^ = 0	7.00 (3)	*p* = .901, η^2^ = 0	4.00 (5)	*p* = .867, η^2^ = 0
Pre-KTx dialysis							
Yes	344	11.00 (9)	*Z* = −.035,	7.00 (4)	Z = −.347,	4.00 (6)	Z = −.134,
No	46	10.50 (7)	*p* = .972, η^2^ = 0	7.00 (4)	*p* = .729, η^2^ = 0	4.00 (5)	*p* = .894, η^2^ = 0
Partnership							
Yes	269	11.00 (9)	*Z* = −.062,	7.00 (4)	Z = −1.113,	4.00 (6)	Z = −.711,
No	128	11.00 (8)	*p* = .951, η^2^ = 0	7.00 (4)	*p* = .266, η^2^ = .003	4.00 (5)	*p* = .477, η^2^ = .001
Donation type							
Living donor	127	12.00 (8)	*Z* = −1.349,	7.00 (4)	Z = −.457,	5.00 (6)	Z = −1.891,
Deceased donor	270	10.59 (8)	*p* = .177, η^2^ = .005	7.00 (4)	*p* = .647, η^2^ = .001	4.00 (6)	*p* = .059, η^2^ = .009

IQR, interquartile range; η^2^, eta-squared.

### Multivariable Analyses for Variables Associated With the SIMS-D

Multiple linear regression analyses were calculated to determine predictors of SIMS-D scores while controlling for sociodemographic variables (**Table 5**). The independent variables explained only 6.5% (*p* < 0.001) of the total variance in the SIMS-D total score. For the linear regression analyses with the SIMS-D “action and usage” and “potential problems” subscales as the dependent variables, respectively 5.4% (*p* < 0.001) and 5.2% (*p* < 0.001) of the total variance was explained. Perceived social support, adherence and age were significantly associated with the SIMS-D total score and the “action and usage” subscale score. Only perceived social support and adherence were significantly linked to the “potential problems” subscale score. The Variance Inflation Factors in the three linear regression analyses were all <1.3 indicating no collinearity between the independent variables.

**Table 5:** Multiple linear regression analyses of variables predicting SIMS-D scores.

Variables	N	β	t	p
**SIMS-D total score as dependent variable**	**374**			
Sex		0.018	0.355	0.723
Age		0.114	2.233	0.026*
Level of education (≤12 years/>12 years)		0.042	0.829	0.407
Perceived social support (F-SozU-K7)		0.188	3.416	0.001**
Adherence (MARS-D)		0.115	2.244	0.025*
Anxiety (HADS-D)		−0.055	−0.984	0.326
**SIMS-D “action and usage” subscale score as dependent variable**	**374**			
Sex		−0.003	−0.057	0.954
Age		0.141	2.761	0.006**
Level of education (≤12 years/>12 years)		0.058	1.145	0.253
Perceived social support (F-SozU-K7)		0.150	2.702	0.007**
Adherence (MARS-D)		0.104	2.012	0.045*
Anxiety (HADS-D)		−0.052	−0.913	0.362
**SIMS-D “potential problems” subscale score as dependent variable**	**374**			
Sex		0.033	0.642	0.521
Age		0.070	1.368	0.172
Level of education (≤12 years/>12 years)		0.021	0.406	0.685
Perceived social support (F-SozU-K7)		0.188	3.395	0.001**
Adherence (MARS-D)		0.105	2.026	0.043*
Anxiety (HADS-D)		−0.049	–0.862	0.389

F-SozU K7, Questionnaire for Perceived Social Support; HADS-D, Hospital Anxiety and Depression Scale; MARS-D, Medication Adherence Rating Scale.

*p < 0.05; **p < 0.01.

## Discussion

This study explored how satisfied KT patients were with the information they had received about their ISM and how satisfaction levels correlated with selected variables. Notable is the general dissatisfaction with ISM information on a broad range of topics. The data indicate that in our sample dissatisfaction was particularly prevalent for items relating to potential problems of ISM, which is in line with the literature. Auyeung et al. (47) reported that health care providers primarily discussed information concerning action and usage of medication with their cardiac in-patients, leaving patients unsatisfied with information about potential problems of the medication. A similar pattern was observed in patients suffering from CKD (19), in patients receiving medication for bipolar disorder (29), in patients diagnosed with rheumatoid arthritis (37), and in a large sample of patients (*n* = 469) suffering from other chronic diseases (30).

The question arises of how aware the health care providers are of this information needs gap and what mechanisms are potentially responsible for explaining this problem. Studies focusing on patient–provider communication suggest that the perception of how thoroughly medical information is discussed can vary considerably when comparing the health care provider’s and the patient’s viewpoint. In a relevant study, physicians overestimated the quantity of provided information: 90% of hospitalized patients stated that they had never been told about side effects of newly prescribed medication, whereas 81% of physicians report describing these effects sometimes (48).

It seems promising to reflect the problem of unmet information needs from both the patient’s as well as the health care provider’s viewpoint. Literature suggests that patients of three different disease groups are very interested in medication information on a wide spectrum of topics (28). However, medication information is sometimes complicated to understand and complex in nature. Research on memory for medical information implicates that patients have difficulty recalling this type of information and often do so inaccurately (49). Another reason for the observed information dissatisfaction could be that patients rarely initiate a discussion about their medication (50). This might collude with physicians having a tendency to ask closed questions (51), thus perhaps discouraging a bilateral discussion about medication information.

A qualitative study by Nair et al. can shed some insight into the reasons of health care providers primarily focusing on action and usage and neglecting other information topics (28). In this study, physicians and pharmacists sensed a certain danger in the amount of provided information, worrying that too much information about medication side effects could be detrimental to the treatment (28). Looking at the data of the present study, only 5% of the reported dissatisfaction with information arose from patients having received too much information; other SIMS studies report a similar dissatisfaction distribution (19, 52). This is further compounded by what the patients desire to know: Ziegler et al. reported that 76.2% of 2,500 adult respondents wanted complete disclosure about potential side effects of medication “no matter how rare” their incidence (53). Moreover, providing this kind of information does not seem to have adverse effects for the patients (28, 54–56). It is important to point out that ISM information satisfaction levels were not associated with the subjective experience of side effects in the present KT sample, making it unlikely that the specific dissatisfaction with potential problems of ISM is a product of actually experiencing side effects. Furthermore, the present study shows that roughly half of the KT patients (40.3–57.1% depending on the item) are currently unsatisfied with information about the topic of ISM side effects. This evidence could help reassure health care providers that this information does not pose a threat to the patient. Finally, other factors impeding information provision are surely rooted in the limitations of the health care systems, where physicians face time constraints in routine care settings (28).

Looking at the individual item level, it is striking that dissatisfaction was greatest for the questions whether ISM will affect a patient’s sexuality or cause drowsiness. Sexuality is of particular interest because of the high prevalence of sexual dysfunctions reported by patients after KTx. Depending on methodology and definition, 48.3–56.9% of male and 44.4–93.4% of female KT patients report symptoms of sexual dysfunction (57–59). Although not uniformly reported (60, 61), a review on this topic concludes that sexual functioning can improve after KTx (62) and could therefore elicit new consultation needs. Literature on the subject suggests that these information needs are not adequately addressed by health care providers, which is corroborated by our results. Muehrer et al. developed the Sexual Concerns Questionnaire and reported that only 60% of a KT sample was given information about sexuality and less than half of these were satisfied with the provided information (63). Sixty-four percent of the KT patients not having received any information were interested in getting more information about this topic (63). This is supported by results from Cabral et al. (64), who found that only 34.6% of female KT patients discussed sexual issues with their physicians even though 73.1% stated this to be an important subject. Sexual functioning is an important KTx outcome factor since sexual concerns (63) and decreased sexual interest/ability (60) are inversely related to quality of life and should thus be made an integral part of the health care provider’s consultation post-KTx (4).

Overall, 57.1% of the KT patients in our sample were dissatisfied with the information they had received regarding drowsiness due to ISM. This warrants special attention, since daytime sleepiness—defined as “difficulty in maintaining a desired level of wakefulness” (65)—is a common phenomenon in KT patients. In three studies conducted by Burkhalter et al. (66–68), about 50% of KT patients reported such symptoms. This is much higher compared to the prevalence of 10.4–33% in the general population (69, 70). Although sleep quality can generally improve after KTx (71), poor sleep quality continues to remain a problem for 30–62% of KT patients (67, 71–74) and seems to contribute to a decreased quality of life (75). Moreover, daytime sleepiness has been shown to be associated with increasing the odds of ISM non-adherence (drug taking component) by 13% (66). The authors hypothesize that daytime sleepiness may pose a non-intentional barrier to adherence and, thus, should be addressed by health care providers (66).

The observed information needs gap potentially could adversely affect adherence behavior and thus entail clinical implications. A first indicator for this is the association between adherence ratings and SIMS-D scores found in our study. Even though not consistently reported in the literature (36, 37), this association has also been observed for other chronic health conditions (21, 25, 29–35, 76). The observed effect size for this association was small in our study; however, Ferguson points out that effect sizes should also be interpreted in light of the possible practical implications (e.g., risk-benefit analysis) of clinical research (77). Comprehensive literature reviews estimate the prevalence of KT patients engaging in non-adherent behavior to range between 22% and 28% (78, 79). The therapeutic window of ISM is very narrow and negative clinical and economic consequences are to be expected for KT patients exhibiting non-adherence to ISM on any scale (79, 80). In this context, all amendable factors promoting adherence at a feasible cost are worthwhile considering due to the magnitude of the described problem. There is evidence that adherence-enhancing interventions for KT patients imparting transplant related information in combination with facilitating emotional and behavioral changes have a beneficial effect on adherence behavior (81). Thus, from a clinical viewpoint, the observed association between non-adherence and dissatisfaction with ISM information received seems highly relevant: information provision is a potential adherence risk factor amendable to change and could therefore be a promising therapeutic target.

The perceived social support in this sample was rather high and significantly associated with the SIMS-D scores. This is a novel finding, since to our knowledge no recent studies have investigated the association between these two variables in a KT sample. Researchers studying social support in the context of health information have previously focused on its association with health literacy: the ability to acquire and understand information relevant for important health-related decisions (82). Patients with low health literacy are prone to hide their limitations due to shame (83), and more than 60% report experiencing little or no social support when it comes to medical information (84). Sleath et al. found in their study with patients taking antidepressant medication that about 32.1% received medication information from friends or family (27). Thus, one pathway of social support influencing ISM information satisfaction levels could be by friends or family being a significant source of medication information. This being a social environment in which patients feel more comfortable to ask health-related questions; the reliability of this source is, however, a different question altogether (76). Indeed, vasculitis patients having received information from different sources with conflicting content (51.3%) were more likely to be non-adherent (85).

The SIMS-D was not correlated with the time passed since KTx and thus does not seem to be a product of time accruing incrementally, which is comparable to knowledge about ISM (9). A longitudinal study over a period of 2 years with liver transplant patients indicated that information needs can change according to the disease trajectory and stay consistently high with regard to treatment process and emotional/physical symptoms (86). Information needs of patients seem to require continuous attention and should thus be a recurrent topic of discussion during the course of treatment.

In the present study higher anxiety levels were correlated with lower levels of satisfaction with ISM information. There is evidence that anxiety can impair memory performance (49). Anxious patients might need more reassurance concerning, e.g., side effects of their medication or, alternatively, less satisfaction with information may increase anxiety levels. This, however, should not be over-interpreted, since the multiple linear regression analyses revealed no significant relationship between the HADS-D anxiety and the SIMS-D.

Sociodemographic variables seem to only play a marginal role with respect to ISM information satisfaction levels. In our study, only age was associated with the SIMS-D subscale score “action and usage” and the SIMS-D total score (positive correlations respectively). This is not consistent with the results of Parham et al. who found an inverse correlation between age and the SIMS subscale score “action and usage” (19) in a sample of CKD patients receiving dialysis treatment. One possible explanation for the association in our sample could be that physicians adopt a more patient-centered interaction style with older patients, as has been shown in previous research (87). This interaction style could possibly allow more room for critical questions and in-depth medical information topics.

### Limitations

This study utilized the widely used self-report measure MARS-D (38) to assess adherence since this tool is efficient, easy to administer, and widely accepted, and there is a validated German version available (41). A variety of methods exist to measure adherence behavior, which all have their advantages and disadvantages. Adherence has been subject to a fair amount of research: although no gold standard has emerged yet for measuring this behavior (88), a review on this subject argues that electronic monitoring devices seem to be the most advantageous method (89). The combination of medical team reports and direct (e.g., variability in immunosuppressive trough levels) and indirect (e.g., self-report measures) methods yield the highest sensitivity for detecting non-adherence (88) and should have ideally been employed to assess adherence. This approach was ruled out in the present study due to cost considerations, thus limiting the validity of the reported non-adherence rates.

Due to the cross-sectional nature of this study, it is not reasonable to assert cause and effect relationships between the SIMS-D and the selected variables; ideally prospective longitudinal intervention studies could elucidate upon the underlying mechanisms.

Finally, the representativeness of the sample can be questioned with respect to the KT patients deciding not to participate. As displayed above, slight, yet significant differences existed in the variables age, time passed since KTx, and donation type between participants and nonparticipants. Perhaps the nonparticipating KT patients have acquired a certain expertise in maintaining their renal allograft, feel comfortable with the existing follow-up structures, and do not perceive a need for the additional support offered in KTx 360°. Furthermore, living donor recipients might be particularly compelled to participate in our study, potentially feeling a special obligation towards their donors to do everything possible to maintain their KT. Nevertheless, it cannot be excluded that there was a sampling bias due to self-selection, which might have influenced our results.

### Strengths

This study used a large consecutive sample of patients after KTx and is the first to evaluate satisfaction with information received about ISM in this population. Specific areas of dissatisfaction with information provided about ISM were identified giving health care providers an insight into the information needs of KT patients. By means of correlation and multivariable analyses, a first step into helping understand ISM dissatisfaction in KT patients was made, hopefully providing a promising foundation for further research.

## Conclusion

Comparable to patients with other chronic illnesses, we found a high prevalence of dissatisfaction with information about ISM in a large sample of KT patients, particularly with regard to information about side effects. KT patients with higher dissatisfaction about ISM information were slightly younger and reported more non-adherence, less perceived social support, and somewhat more anxiety. Even though the effect sizes for these associations were small, the observed dissatisfaction with ISM information is important to address due to the possible implications for adherence behavior. From a risk–benefit point of view, providing information entails little harm for the patient and comes at a feasible cost for the health care system with potential benefits for adherence behavior; it is also a contentious issue since it is the legal duty of health care providers to educate patients comprehensively (10). Current information provision seems to have a generic patient in mind, not adequately taking the individual patient’s viewpoint and needs into account. A possible remedy for the reported shortcomings is good communication practices between health care providers and patients, for which both sides share a joint responsibility. Health care providers could coordinate their roles more smoothly in the context of information provision and provide a welcoming consultation atmosphere. This could enable bilateral discussions about critical ISM questions to help overcome the discussed information needs gap. The key to good prescription practice would thus require health care professionals to collaborate proficiently and tailor the provided information to meet the personal needs of the individual patient (19, 21, 76).

## Data Availability Statement

The datasets generated for this study are available on request to the corresponding author.

## Ethics Statement

This study was carried out in accordance with the recommendations of the ethics committee of the Hannover Medical School with written informed consent from all subjects. All subjects gave written informed consent in accordance with the Declaration of Helsinki. The protocol was approved by the ethics committee of the Hannover Medical School (number 3464-2017).

## Author Contributions

LP, MS, MZ, and UT designed the trial, and LP and MS obtained research funding. LS was essential in the recruitment process of the study. FK, MN, and MB-H collected the data for this analysis. FK and MZ wrote the first draft of this paper, which has been critically revised by all coauthors. All authors have read and approved the final version of the manuscript.

## Funding

The study is supported by a grant from the Federal Joint Committee of the Federal Republic of Germany under the number 01NVF16009 (Trial registration: ISRCTN29416382).

## Conflict of Interest Statement

The authors declare that the research was conducted in the absence of any commercial or financial relationships that could be construed as a potential conflict of interest.

The handling editor declared a past co-authorship with one of the authors MZ.
